# Collective Investigation of Dental Diseases

**Published:** 1885-03

**Authors:** 


### Collective Investigation of Dental Diseases.

London, December 15, 1884.

Collective investigation of disease amongst medical practitioners has become an accomplished fact.

The British Medical Association, with characteristic zeal and energy suggested the desirability of collecting brief but accurate statistics of various diseases. These statistics were to be furnished by busy men in busy practice, were to be entered upon cards or forms printed in such a way as to require of the medical man but little time or labor, for he would have only to refer to his note book for cases and to jot down his answers.

The mass of material thus accumulated was placed in the hands of a competent committee who gleaned results which were subsequently published with hints to those who were willing to prosecute the research further. By giving out some medical *crux*, and issuing a card for each, any and every medical man who cared could contribute his mite to the general fund of information.

The success which crowned the labors of these collective investigations has naturally enough led to the extension of the field of research. Addresses delivered at the Congress, held at Copenhagen, were instrumental in initiating an International Collective Investigation, an organization which will, unless its founders are over-sanguine, lead to very valuable results. It is surprising that this useful form of investigation has not been extended to special departments, and notably to Dental Medicine and Dental Surgery. Collective investigation among dentists should lead to the accumulation of much information. There are so many questions still undecided in the practice and pathology of dentistry, so many which would be vastly elucidated could the experience and research of the majority of the thoughtful dentists be obtained. This method of gathering results together admits of peculiarly easy treatment by the "timeless toiler." So slight a sacrifice of leisure is entailed upon the recorder of his experience that it seems probable, were the investigation once floated, that dentists would be at least as willing as are medical men to render assistance. So much has been written, and so much has been said about caries attacking teeth that the phrase " dental caries," is a household expression, but how very slender is the grasp we have upon its pathology, and how shallow are even the most specious arguments advanced in support of the many theories advanced in explanation of dental caries. Or again, if we take pyorrhœa alveolaris, a condition so familiar, and yet one so indifferently understood, that its treatment must be termed at best, purely an empyrical one, while the true conditions at work upon the diseased tissues are yet shrouded in doubt and uncertainty. Questions such as how far early drugging of children directly leads to dental disease and deformity, and the whole field of practical dentistry would undoubtedly be benefitted by a well conducted collective investigation. In urging the utility of this scheme upon the dental profession, it seems to us we are indicating a means of rescuing from loss a really valuable mass of facts. But what is everybody's business is nobody's business! To make the research of any practical value, the subjects should be chosen by competent judges, by men who have learned in the school of experience just what we know, and of what we are ignorant, and further should be able to select really practical, useful subjects for research. Recondite conditions, however interesting to the pathologist, may be of little or no practical value outside his narrow circle, and so would call for but little comment from the average practitioner.

How far the inertia which forms so striking a trait of some of the dental profession, would swamp any attempt in this direction only the course of events will show. The more exact methods of research and greater precision of teachers are rapidly turning out practitioners who could do the duties of members of a collective investigation society, and could perform them well. The tendency is to forget the minutiæ of one's early lessons, and this tendency would be powerfully resisted were men to habitually report experiences, modes of treatment, etc.

